# Draft Genome Sequence of Hypervirulent and Vaccine Candidate *Streptococcus* *suis* Strain SC19

**DOI:** 10.1128/genomeA.01484-16

**Published:** 2017-01-19

**Authors:** Lin Teng, Xingxing Dong, Yang Zhou, Zhiwei Li, Limei Deng, Huanchun Chen, Xiaohong Wang, Jinquan Li

**Affiliations:** a College of Food Science and Technology, Key Laboratory of Environment Correlative Dietology, Huazhong Agricultural University, Wuhan, Hubei, People’s Republic of China; b Department of Animal Sciences, University of Florida, Gainesville, Florida, USA; c Department of Infectious Diseases and Pathology, University of Florida, Gainesville, Florida, USA; d College of Fisheries, Huazhong Agricultural University, Wuhan, Hubei, People’s Republic of China; e State Key Laboratory of Agricultural Microbiology, Huazhong Agricultural University, Wuhan, Hubei, People’s Republic of China; f Jiangsu Key Laboratory of Zoonosis, Yangzhou University, Yangzhou, Jiangsu, People’s Republic of China

## Abstract

*Streptococcus suis*, a zoonotic bacterium found primarily in pigs, has been recognized recently as an emerging pathogen of humans. Herein, we describe the genome of *Streptococcus suis* strain SC19, a hypervirulent and vaccine candidate strain isolated from a pig amid the 2005 outbreak in China.

## GENOME ANNOUNCEMENT

*Streptococcus suis* is an important pathogen associated with a variety of life-threatening infections, including meningitis, endocarditis, arthritis, septicemia, and even sudden death in pigs (1). *S. suis* is responsible for significant worldwide economic losses in the pig industry (2). Among the 33 known serotypes, *Streptococcus suis* serotype 2 is considered to be the most prevalent and virulent. Although only sporadic cases of *S. suis* infection have been reported in humans (3), two outbreaks (Jiangsu in 1998 and Sichuan in 2005) initiated in China have raised considerable concerns among public health and food safety professionals in recent years (4).

*S. suis* serotype 2 strain SC19 (also known as SC-19, 05ZYS, or ZYS) was isolated from the brain of a diseased piglet during the Chinese *S. suis* outbreak in 2005. SC19 has been utilized as a prototypic virulent *S. suis* strain in multiple studies. Foremost, immunoproteomic analysis of strain SC19 identified 34 cell wall-associated proteins, indicating alternative antigens for the further development of *S. suis* vaccines and diagnostic markers (5). Second, more than 15 virulence factors, such as GlnA, CiaRH, PerR, SsPepO, HP0197, and SspA, were characterized from this strain (6–8). Third, to increase the knowledge of the pathogenesis of meningitis, septicemia, and pneumonia in pigs caused by *S. suis* 2, gene expression profiles of peripheral blood mononuclear cells (PBMC), porcine monocyte-derived dendritic cells (MoDC), human monocytic cells (THP-1), as well as brain and lung tissues upon infection with strain SC19 were investigated by microarray (9–11). Fourth, several SC19-based live vaccine candidates were developed, among which at least one patent has been authorized in China (our unpublished data). SC19, as a hypervirulent strain, has been broadly used to evaluate the protective efficacy of vaccine candidates. However, in accordance with the scope of this announcement, the genomic composition of SC19 is poorly understood. Therefore, a draft genome sequence of *S. suis* strain SC19 has been compiled to further support the development of new commercial vaccines and diagnostic markers.

The genome was sequenced on the HiSeq platform (Illumina, San Diego, CA, USA) using a paired-end library with a 150-bp read length. The *de novo* assembly strategy was performed through Edena version 3.131028 (12). The assembly generated 23 contigs with 2,073,313 bp (*N*_50_, 223,803 bp), followed by genome annotation using the RAST genome annotation server (version 2.0) (13–15). The genome of SC19 has 2,008 coding sequences, five rRNAs, 45 tRNAs, and a G+C content of 41.0%. The genome encodes the tetracycline resistance protein TetM and presents previously described virulence gene candidates, such as *salK*, *salR*, *nisK*, *nisR*, *sly*, *ssnA*, *endA*, *scpA*, *epf*, *fhb*, *ide*_*Ssuis*_*, nudp*, *mrp*, *ssadS,* and *igA1*, and an 89-kb pathogenicity island (89 K PAI). Since SC19 has a clear origin and significant empirical evidence of hypervirulence (5, 6), it can serve as a prototypic *S. suis* strain. Genomics Insights of SC19 will support comparative genomics and pathogenicity analyses, therapeutic and vaccine development, and will ultimately help protect the swine industry and human health.

### Accession number(s).

This whole-genome shotgun project has been deposited in GenBank under the accession no. MNPY00000000. The version described in this paper is the first version.
